# Evaluating the Effectiveness of the Computer-Based Education Platform, Pharmacy5in5, on Pharmacists’ Knowledge of Anticholinergic Toxicity Using a Randomized Controlled Trial

**DOI:** 10.3390/pharmacy10010008

**Published:** 2022-01-01

**Authors:** Rand Hussein, Zhoushanyue He, Julia Bareham, Tejal Patel, Rosemary Killeen, Kelly Grindrod

**Affiliations:** 1 School of Pharmacy, University of Waterloo, Waterloo, ON N2L 3G1, Canada; rnyhussein@uwaterloo.ca (R.H.); tejal.patel@uwaterloo.ca (T.P.); r2killeen@uwaterloo.ca (R.K.); 2 Department of Statistics and Actuarial Science, University of Waterloo, Waterloo, ON N2L 3G1, Canada; hezhoushanyue@gmail.com; 3 RxFiles Academic Detailing, College of Pharmacy and Nutrition, University of Saskatchewan, 107 Wiggins Rd, Saskatoon, SK S7N 5E5, Canada; julia@rxfiles.ca

**Keywords:** computer-based education, knowledge, anticholinergic toxicity, pharmacists, education, distance

## Abstract

Background: Computer-based education has been widely implemented in healthcare professional development education. However, there has been little examination of the potential for computer-based education to enhance pharmacists’ knowledge. This study aims to assess the effectiveness of computer-based education on improving pharmacists’ knowledge compared to printed education material. Methods: This study was a web-based randomized controlled trial. Participants were randomly allocated to either an intervention group where they had access to the computer-based education module on Pharmacy5in5.ca or to a control group where they had access to printed educational material. Knowledge gain was assessed using a pre- and post-knowledge test. Results: A total of 120 pharmacists were recruited and 101 completed the post-knowledge test (50/60 in the intervention group; 51/60 in the control group). Both groups showed a significant increase in knowledge gain (intervention group: pre-test mean score 19.35 ± 3.56, post-test mean score 22.42 ± 3.812, *p* value < 0.001; control group pre-test mean score 19.22 ± 3.45, post-test mean score 23.29 ± 3.087, *p* value < 0.001). However, the difference in knowledge change was not significant between the two groups (22.42 vs. 23.29, *p* value = 0.333). Conclusions: In this study, a computer-based education module enhanced pharmacists’ knowledge to a similar degree to printed education material. Efforts should be made to provide computer-based education as an option to support pharmacists’ professional development.

## 1. Introduction

Many commonly prescribed and over-the-counter medications such as antihistamines and tricyclic antidepressants have anticholinergic properties, contributing to a wide range of side effects such as dry mouth, delirium, and urinary retention [1]. The aging population, which is growing in Canada, is particularly vulnerable to these side effects due to comorbidities, polypharmacy and potential drug–drug interactions [2,3,4]. Using one anticholinergic medication may result in problematic side effects, but using multiple anticholinergic medications can create a cumulative anticholinergic burden leading to anticholinergic toxicity [5,6]. Further, a 2015 systematic review by Salahudeen et al. [7] highlighted that a higher anticholinergic burden leads to a decline in cognitive ability and physical function among older adults. Moreover, anticholinergic toxicity can lead to emergency department admissions [4,5].

Pharmacists, as highly accessible healthcare professionals, are in an ideal position to identify patients at high risk of developing anticholinergic toxicity, and to provide or recommend safer alternatives to reduce anticholinergic burden. In particular, patients’ easy access to over-the-counter medications with anticholinergic properties, as well as, the high prevalence of inappropriate use of anticholinergic medications [3], makes it important for pharmacists to review anticholinergic burden in their patients. Moreover, pharmacies often have the most comprehensive medication lists of all the healthcare organizations, especially for patients that use a single pharmacy for all of their medication needs. This permits pharmacists to comprehensively examine the anticholinergic burden their patients may be exposed to through the use of both prescription and non-prescription drug use. A 2020 systematic review by Nakham et al. found that medication reviews conducted by pharmacists decreased anticholinergic burden effectively [8]. Given the significant role of pharmacists in optimizing anticholinergic medication use [8,9,10], it is necessary to provide adequate training and support in this area.

Different types of educational interventions are available for professional development for pharmacists. For example, passive interventions such as printed educational material are commonly used [11]. A study by Kouladjian et al. [12] reported that journal articles are appropriate for educating pharmacists on estimating drug burden index, a measure of the total exposure to medications with anticholinergic and sedative properties. However, pharmacists may prefer more active interventions such as computer-based education [13,14,15]. Compared to printed material, computer-based education can increase convenience by delivering content at any time and from anywhere, while allowing self-paced learning. Moreover, it increases users’ interactivity and engagement with the material [16,17].

The ability of computer-based learning to improve knowledge has been well established for nurses and physicians, however, there is limited evidence evaluating its use among pharmacists [18,19]. Moreover, most studies targeting pharmacists focus on pharmacy students rather than experienced pharmacists [20,21,22,23]. Furthermore, there is limited research assessing pharmacists’ knowledge on anticholinergic toxicity using a computer-based educational platform. Hence, the aim of this study is to assess the effect of a computer-based educational platform, Pharmacy5in5, on pharmacists’ knowledge of anticholinergic toxicity compared to using standard printed educational material.

## 2. Methods

### 2.1. Study Design

**Figure 1:**
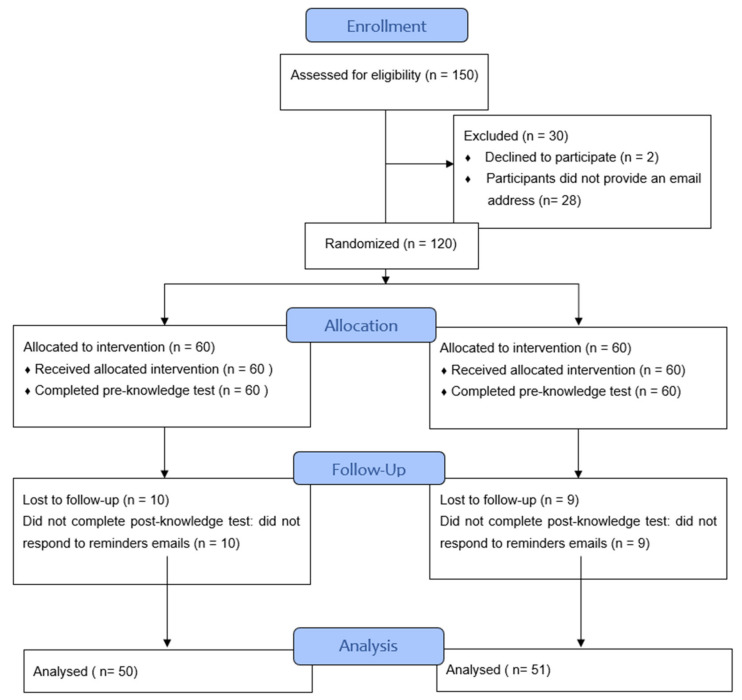
Study flowchart.

A two-arm, parallel web-based randomized controlled trial (RCT) was used to assess the effectiveness of the Pharmacy5in5.ca platform on pharmacists’ knowledge. Ethics approval for this study was obtained from the University of Waterloo Ethics Committee (ORE # 42710). The RCT was conducted in accordance with CONSORT-EHEALTH checklist [24]. Participants were randomly allocated to the intervention group or the control group as shown in the study flowchart below (Figure 1).

### 2.2. Recruitment

All registered users of Pharmacy5in5.ca in Ontario (*n* = 9314) were invited to participate in the study via email. In the email, users received a consent form and a link to a survey to screen for eligibility according to inclusion/exclusion criteria. For this study, inclusion criteria included being a registered user of the Pharmacy5in5. platform and a licensed or registered pharmacist practicing in the province of Ontario, Canada. Exclusion criteria included Pharmacy5in5.ca registered users who were pharmacy technicians, pharmacy students, pharmacy technician students, or unlicensed pharmacists. A total of three recruitment emails were sent to increase response rate [25,26].

### 2.3. Study Procedure

All potential participants were asked via email to complete an online 26-question pre-test and demographic survey. Participants who completed the baseline assessment were assigned a unique study identifier and randomly allocated to one of the two study arms. Participants allocated to the intervention group were sent a second email with a link to the Pharmacy5in5 platform, with access granted to an Anticholinergic Toxicity module. Participants were given one week to complete the one-hour Anticholinergic Toxicity module on Pharmacy5in5.ca. This module was designed specifically for this trial and had not been released to other platform users (only users enrolled in the intervention arm had access to this new module to reduce the risk of contamination [27]). Users had the freedom to access all module components in any order they chose. No additional content development or refinement was performed on the platform or modules while conducting the trial. The intervention group had continuous access to the Pharmacy5in5 platform, which is available on computers, tablets, and smartphones. Given that recruited users were already registered users of the Pharmacy5in5 platform, no training was provided before or during the intervention on how to use the platform. Participants allocated to the control group were instructed via a second email to review printed education material, which included a paper-based decision aid, over the same one-week time frame.

After one week of accessing the Pharmacy5in5.ca module or the print education material, all participants were invited to complete a post-test via email. In the email, participants in the intervention group were asked to complete the module quizzes before taking the post-test. The post-test quiz included the same questions as the pre-test, but in a different order to assess knowledge gain. To access the post-test quiz, participants were asked to enter their study ID and were instructed to answer the questions to the best of their ability and not to consult other materials while taking the quiz. Throughout the study, up to four reminder emails were sent to prompt participants to complete the post-knowledge test.

### 2.4. Interventions

#### 2.4.1. Computer-Based Educational Platform

Pharmacy5in5 is a computer-based education intervention that is freely available to all pharmacy professionals in Canada. The platform aims to accelerate the adoption of best practices by pharmacy professionals. On registration, users provide their demographic information along with consent to use their non-identified data for research purposes. The platform was developed through three cycles of user testing with pharmacists, pharmacy technicians, and students, where users were asked to interact with the platform while explaining their impressions, actions, and any areas of confusion. Users also completed the Systems Usability Scale (SUS) as a validated measure of usability.

Pharmacy5in5 is designed to regularly release modules that cover five take home messages about a clinical or pharmacy practice topic. Each module is created by a team of experts to provide real-world examples of how evidence, guidelines and practice tools can be used in daily practice. The platform also provides much needed information about trending topics. For example, in the recent “COVID-19” module, users were provided with latest evidence regarding the use of COVID-19 vaccines during pregnancy and breastfeeding. Each module has the following design components: One fast facts quiz with immediate feedback; six case-based quizzes, with delayed feedback [28]; peer comparison; self-reflection questions to self-report behaviours; and multimedia resources

- Quizzes: each module consists of one “Fast Fact quiz” and six “Case-based quizzes” that are based on the module learning objectives. The “Fast Fact quiz” tests users’ basic knowledge about the clinical topic, while “Case-based quizzes” test users’ knowledge and behaviour through helping a fictional pharmacist make the optimal decision in cases based on real-life scenarios. Questions used are multiple choice or true/false.
- Feedback: in each module, online quizzes are followed with feedback on users’ performance. Each “Fast Fact quiz” provides users with immediate feedback after each question to let them know if they got the right answer. “Case-based quizzes” provide delayed feedback after the user finishes the whole quiz series, along with the correct answer, rationale, and links to useful resources underneath each question.
- Peer comparison: this feature aims to compare a user’s performance to an average user and provides feedback on user progress. Peer comparison also rewards users who outperform an average user, and motivates users who perform below average.
- Self-reflection: after each case-based quiz, users are asked to answer a question about their past behaviour. Self-reflection questions are structured as follows: “in the past 3 months have you/did you…”. This feature allows users to self-report their behaviours, which can help detect any changes in users’ behaviour before and after taking the module.
- Multimedia resources: there is a set of specific resources created for each module, including: flashcards, infographics, and short videos. These multimedia resources highlight the key take home message for each clinical topic and can be shared on social media.

The platform was designed using a combination of the Theoretical Domains Framework (TDF), the COM-B (capability, opportunity, motivation, and behaviour) model, and the Behaviour Change Wheel (BCW), to understand factors that influence pharmacist behaviour. The TDF includes 14 domains for potential determinants of behaviour (e.g., knowledge, skills, beliefs about capabilities, etc) [28]. The COM-B model demonstrates how an individual’s capability, opportunity, and motivation impact behaviour, and can be linked back to specific TDF domains [29]. Based on the COM-B, Pharmacy5in5 aims to change pharmacist behaviour by targeting pharmacist capability. This corresponds to TDF behaviour change domains such as knowledge, skills, decision processes, and behavioral regulation [29]. The BCW shows how the COM-B domains can be targeted by a variety of behavioural interventions and policies [30]. Pharmacy5in5 uses three behavioural interventions, including modelling (learning through imitation), education (gaining knowledge), and training (gaining skills).

#### 2.4.2. Printed Materials

The printed education material used for this study was provided as an 11 pager PDF document and included two references: (1) RxFiles: Reference List of Drugs with Potential Anticholinergic Effects which is a paper-based decision aid [31]; and (2) Anticholinergic Toxicity review by Broderick et al., 2020 [5]. These two references were used in the development of the Pharmacy5in5 online module content. The RxFiles drug charts are unique decision aids used by Canadian pharmacists and physicians in practice. The charts provide evidence-based information about drugs to help providers make therapy decisions [32].

### 2.5. Anticholinergic Toxicity Module Development and Validation

**Figure 2:**
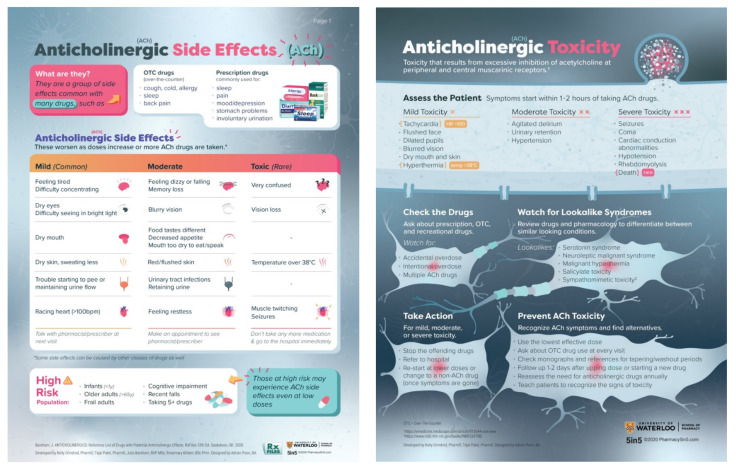
Anticholinergic Toxicity module educational infographics.

**Figure 3:**
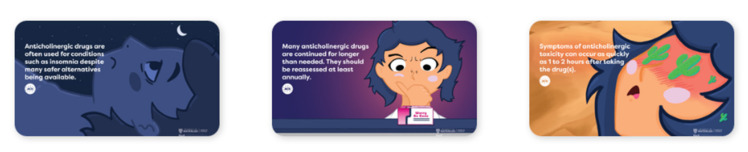
Anticholinergic Toxicity module flashcards.

To create the *Anticholinergic Toxicity* module, the Pharmacy 5in5 content developers started by drafting learning objectives (Box 1). Next, the learning objectives were used to develop two educational infographics (Figure 2), one short animated video (1:06 min long), and five flash cards (Figure 3). The content developers also developed an introductory immediate feedback quiz and six case-based delayed feedback quizzes to address the learning objectives. Next, all the module components were reviewed through two rounds with a panel of three experts. In the first round, the content of the module was shared with the panel via email to gather comments and critiques for each question and all multimedia resources, and modifications were made accordingly. A total of seventeen questions were modified and the two infographics were clarified based on expert feedback. For the second round, the full module was shared after modifications and 12 questions were reworded slightly as per expert feedback. Before launching the study, the module was piloted with five practising pharmacists to assess any concerns with readability and clarity of wording.

Box 1 ***Anticholinergic Toxicity* module learning objectives**

1. Recognize anticholinergic side effects when assessing patients, including anticholinergic toxicity.
2. Identify higher risk anticholinergic drugs, including additive effects.
3. Classify patients according to their risk for serious anticholinergic side effects.
4. Identify safer alternatives for indications where anticholinergic drugs are commonly used.
5. Implement a plan to switch an anticholinergic drug to a safer alternative, deprescribing, tapering, switching, and incorporating washout periods.

### 2.6. Outcome Measure

The main outcome measure was the difference in the post-test quiz score between the intervention and control group using pre-post validated knowledge tests. A secondary outcome measure was the change in the intervention group’s and control group’s quiz scores, before and after the intervention.

### 2.7. Sample Size Calculation

With a power of 80% and a two-tailed alpha of 0.05, an estimated 45 users were needed for each group to show a 5% difference in knowledge between the two arms post-test scores (standard deviation 1.23 based on a previous pilot study, 2-sided test, *p* < 0.05, power of 80%). Taking into consideration the high dropout rate with internet-based interventions [24,33], we enrolled 120 participants to account for an expected 25% dropout rate.

### 2.8. Randomization and Blinding

After enrollment, eligible participants were randomised using a 1:1 allocation ratio via a computer-generated random number by an independent research member who was not involved in the data analysis. Given the nature of the study, it was not feasible to blind the participants. However, only the primary investigator was aware of the allocations. The study team was blinded to allocation until the analysis was completed.

### 2.9. Data Analysis

Average scores were computed for the primary outcome (knowledge score out of 29) and reported as mean ± SD. The normality of pre- and post-knowledge test scores were tested using Kolmogorov–Smirnov and Shapiro–Wilk test. As the data was found to be skewed, nonparametric tests were used to compare the outcomes between the control and intervention groups using Mann–Whitney U test and Wilcoxon Signed ranks tests to compare within groups at before and after the intervention [18]. Additional analyses included assessing differences in baseline sociodemographic factors between the control and intervention groups, and between respondents and non-respondents using Chi-square and Fisher’s exact test. *p* < 0.05 was considered statistically significant. Statistical analysis was performed using SPSS version 27 statistical package (IBM Corp, New York, NY, USA) [34].

### 2.10. Knowledge Test Development and Validation

#### 2.10.1. Knowledge Test Development

To assess if the use of the platform results in knowledge gain, a knowledge test was developed as outlined by Case and Swanson [35]. The first draft of knowledge test included a total of 33 questions that were developed based on the module learning objectives. The knowledge test questions were different from the questions in the module quizzes and included a combination of clinical vignette questions and standard questions. The questions were constructed to address Blooms’ Taxonomy lower and higher order thinking levels. First, the knowledge test was reviewed by two senior content developers who were asked to code each question to the corresponding learning objective and Bloom’s level to ensure that there were at least three questions addressing each learning objective as well as an equal number of questions assessing Bloom’s Taxonomy lower and higher order thinking levels.

#### 2.10.2. Knowledge Test Validation

The knowledge test was validated with geriatric pharmacotherapy experts in two rounds. In the first round, the experts were asked to rate each question in terms of relative importance to the stated learning objectives (1 = Not relevant; 2 = Somewhat relevant; 3 = Quite relevant; 4 = Very relevant). They were also instructed to provide their feedback and comments to improve the questions. Agreement among experts was used to calculate the content validity index for each item (item content validly index [i-CVI]), where items with i-CVI values equal or less than 0.79 required revisions, and i-CVI values less than 0.70 were eliminated [36,37]. The first round of content validation was conducted with seven experts who reviewed the 33 questions. After revision, the second draft was composed of 26 questions (23 multiple choice questions; 3 open-ended questions). Seven questions were deleted because they were considered repetitive or did not fit with any of the learning objectives. A total of 12 questions were revised or reworded to improve clarity. Of the 33 questions, only six questions had a low level of agreement among experts (i-CVI = 71.4) (see Appendix A).

For the second round, the revised version of the knowledge test with 26 questions was shared with the experts to gather their feedback and collect any additional comments. Only three experts agreed to participate in the second round. Minor grammatical modifications and comments were recommended for seven questions.

The knowledge test internal consistency and item difficulty index were assessed using the first 50 responses [38]. Items with an item difficulty index less than 0.2 or higher than 0.8 were eliminated or revised [37]. The internal consistency of the 26 items, using Cronbach’s alpha (α), was 0.73. In terms of the item difficulty index, four items had a difficulty index of 0.9 or above and were deleted to allow for differentiation in learning. Two items had a difficulty index less than 0.2, the two items were retained and one of them was revised for clarify (10.a). The final draft of the knowledge test included 22 items:19 multiple-choice questions (MCQs) and 3 open-ended questions (see Appendix B). The final score was calculated out of 29 as follows: The score of the MCQ questions (out of 19) was automatically generated via Qualtrics, and the score for the open-ended questions (out of 10) was calculated based on a pre-validated grading rubric.

## 3. Results

### 3.1. Demographics of Participants

All pharmacists practising in Ontario who were registered users of Pharmacy5in5.ca were invited to participate in the trial. A total of 120 users agreed to participate and were randomized to the intervention group (60 participants) and the control group (60 participants). No statistically significant differences were found between the demographic characteristics of the two study groups (Table 1). Most respondents were female (78.3%), held a bachelor’s degree (63.3%), received their training in Canada (72.5%), had more than 10 years of pharmacy practice experience (58.3%), and were practising in community pharmacies (70.8%). In terms of previous training, 96% of participants denied receiving any training related to anticholinergic toxicity in the past 12 months.

### 3.2. Loss to Follow Up

Of the 120 pharmacists who were randomized, all completed the pre-test and 101 (84.2%) completed the post-test. In particular, the post-test was completed by 83.3% (50/60) of the intervention group, and 85% (51/60) of the control group. There was no significant difference in baseline demographics of respondents and nonrespondents in the intervention group. There was no significant difference in the pre-test scores between respondents and nonrespondents in the intervention group (pre-test mean score 19.64 ± 3.49; pre-test mean score 17.9 ± 3.66; *p* value = 0.159). Similarly, in the control group, there was no significant difference in baseline demographics of respondents and nonrespondents, and no significant difference in the pre-test scores between respondents and nonrespondents (pre-test mean score 19.22 ± 3.37; pre-test mean score 19.22 ± 4.08; *p* value = 0.699)

### 3.3. Assessment of Access to the Anticholinergic Toxicity Module

Out of the 60 pharmacists allocated to the intervention group, 41 (68.3%) accessed the module and completed all seven quizzes, one user completed six out of the seven quizzes, one user completed five out of the seven quizzes, and 15 (25%) did not access the module at all. Of the 15 who did not access the module, seven completed the post-test.

On average, pharmacists in the intervention group spent 40 min to complete the quizzes of the module in a single session. Response data also showed that 15/43 (34.8%) of pharmacists completed the knowledge post-test within one day of completing the module quizzes, 26/43 (60%) completed the knowledge post-test after one week of completing the module, and 2/43 (4.6%) completed the knowledge test after two weeks of completing the module quizzes.

### 3.4. Knowledge Test

As shown in Table 2, the mean score for the knowledge pre-test was 19.35 ± 3.56 (67%) in the intervention group and 19.22 ± 3.45 (66%) in the control group. There was no significant difference in the knowledge pre-test mean scores between the two groups (Mann–Whitney U: Z value = −0.018, *p* = 0.985). The mean score of the knowledge post-test increased significantly to 22.42 ± 3.812 (77%) in the intervention group and 23.29 ± 3.087 (80%) in the control group (Wilcoxon Signed Rank: Intervention group: Z value = −4.690, *p* < 0.001; Control group: value = −5.180, *p* < 0.001). However, there was no significant difference between the two study groups in the knowledge post-test (Mann–Whitney U: Z value = −0.968, *p* = 0.333).

In terms of individual questions, the two questions that had the lowest performance were those that addressed classifying patients according to their risk for serious anticholinergic side effects. In particular, only 16% of the participating pharmacists were able to identify that low dose doxepin (3 mg) is not a risk factor for serious anticholinergic side effects. Moreover, only 16.8% recognized that Parkinson’s disease is not a risk factor for serious anticholinergic side effects. See Appendix C and Appendix D for subgroup analysis of post-test knowledge questions.

## 4. Discussion

This randomized controlled trial assessed Ontario pharmacists’ knowledge of anticholinergic toxicity after completion of a computer-based education module. One of the key findings is that the computer-based education module improved pharmacists’ knowledge significantly (*p* < 0.001). This result is consistent with previous studies conducted among nurses and physicians [39,40,41]. Moreover, the study showed that there was no difference between a paper-based decision aid by RxFiles and a computer-based education module in improving pharmacists’ knowledge of anticholinergic toxicity, as there was no significant difference in post-test scores between the study groups (*p* = 0.208). Surprisingly, the study found that the topic of anticholinergic toxicity is not commonly addressed in pharmacy professional trainings with only 4% of participants reporting having received training related to the topic in the past 12 months. Given that anticholinergic medications are problematic in an aging population, and that the primary users of medications are the elderly, pharmacists are required to have the ability to address any drug-related problems that arise with the use of these medications. The Pharmacy5in5 platform offers busy pharmacists the opportunity to easily access modules via an online platform and test their knowledge and receive feedback on their behaviours.

This study contributes to existing literature with few studies comparing the effect of computer-based education to printed educational material among pharmacists. In a recent scoping review of the effect of computer-based education on health care providers’ knowledge skills and behaviour, a total of 14 studies assessed knowledge gain [19]. Only one study targeting pharmacists was identified. Nesterowicz et al. [18] compared computer-based education to a two-hour on-site session on improving pharmacists’ knowledge of blood pressure monitoring. The authors assessed knowledge gain after six months. Similar to our study, the increase in knowledge in both groups was significant by 29% in the intervention group and 27.2% in the control group, but the groups did not differ significantly, indicating that online learning is as effective as on-site learning. Pharmacists in the study had a low pre-test scores indicating more room for improvement in their knowledge. Despite the difference in topic addressed, this indicates that computer-based education could promote knowledge retention among pharmacists and that there should be a move towards readily available, easily accessible, self-directed computer-based education, especially during the COVID-19 pandemic.

While this study indicated that computer-based education is an appropriate intervention to educate pharmacists on anticholinergic toxicity, the knowledge gained is not necessarily translated into clinical practice [42]. A successful behaviour change intervention should address barriers and facilitators to promote practice change [43]. As such, computer-based education could alter pharmacists’ behaviour by addressing unique barriers to reducing anticholinergic burden. A 2021 systematic review by Stewart et al. [44] identified a number of barriers among pharmacists including lack of confidence in skills and uncertainty about the need for reducing anticholinergic burden. The *Anticholinergic Toxicity* module used in this study could help pharmacists overcome these barriers through the six case based quizzes to build confidence, and the animated video emphasizing the consequences of anticholinergic toxicity [45]. Therefore, computer-based education could be tailored to address barriers using different design components, making it a promising intervention to optimize the use of anticholinergic medications

### 4.1. Strengths and Limitations

A strength of this study was a low dropout rate of 15.8% despite being conducted during the COVID-19 pandemic, as compared to other studies among healthcare professionals that reported dropout rates of 31% [46] and 47% [47]. This suggests that there is high feasibility for conducting online interventions with pharmacists. The dropout rate in both the intervention and control groups was similar, which suggests that both the printed and computer-based education platform were desirable. A second strength of the study was the triangulation of the post-test knowledge scores with the usage of the platform, where 86% of the users who completed the post-test had completed the module. Another study strength was the low level of contamination bias as only 1 out of 101 users reported that they knew other pharmacists who participated in the study, and 9 users were unsure. However, all users reported that they did not discuss their group allocation with a colleague, nor knew whether they were assigned to a different group. A potential limitation is that the content of the education modules was focused on a single topic (anticholinergic toxicity), with a plan for future work to assess other pharmacy practice-related content to demonstrate the generalizability of the effect of computer-based education on pharmacists’ knowledge. Another limitation, is the limited ability to control or monitor the use of additional resources while completing the post-tests. Moreover, there was limited ability to confirm whether to the control group users completed the paper-based material before completing the post-knowledge tests. Another potential limitation is selection bias, with the possibility that only pharmacists who were interested in the topic agreed to participate in the study [48].

### 4.2. Practical Implications and Future Research

This study provided much needed information about the effectiveness of the computer-based education platform in improving pharmacists’ knowledge. The results also act as a feasibility study for a future study of how the website supports behaviour change and practice outcomes. Pharmacy5in5 is anticipated to be a highly impactful platform with the potential to advance the field of professional continuing education for pharmacists.

## 5. Conclusions

This study evaluated the effectiveness of a computer-based education module compared to a paper-based education in improving pharmacists’ knowledge of anticholinergic toxicity. The study highlighted that computer-based education can improve pharmacists’ knowledge similarly to printed education material. Further research is needed to assess the potential of computer-based education in improving pharmacists’ knowledge in non-clinical topics such as ethics and policies, as well as assessing if knowledge gained from computer-based education is retained and implemented in pharmacy practice.

## Figures and Tables

**Table 1:** Demographics of the control group and intervention group participants.

Demographics	Intervention Group (*n* = 60)	Control Group (*n* = 60)
Years of pharmacy practice experience		
Less than 1 year	3 (5%)	6 (10%)
1–5 years	14 (23.3%)	13 (21.6%)
6–10 years	9 (15%)	5 (8.3%)
11–20 years	12 (20%)	15 (25%)
More than 20 years	22 (36.6%)	21(35%)
Gender		
Woman	44 (73.3%)	50 (83.3%)
Man	13 (21.6%)	8 (13.3%)
Prefer not to disclose	3 (5%)	2 (3.3%)
Location of qualifying pharmacy training (e.g., BScPharm, entry level PharmD)		
Canada	45 (75%)	42 (70%)
United States	3 (5%)	5 (8.3%)
Outside North America	12 (20%)	13 (21.6%)
Highest level of education		
Bachelor	38 (63.3%)	38 (63.3%)
Entry-level PharmD	10 (16.6%)	11 (18.3%)
Masters	4 (6.6%)	5 (8.3%)
Postgraduate PharmD	6 (10%)	3 (5%)
PhD	1 (1.6%)	1 (1.6%)
Other	1 (1.6%)	2 (3.3%)
Primary site of practice		
Community: Independent pharmacy	20 (33.3%)	22 (36.6%)
Community: chain or franchise	22 (36.6%)	21 (35%)
Hospital in-patient	9 (15%)	8 (13.3%)
Primary care clinic	4 (6.6%)	3 (5%)
Other	5 (8.3%)	6 (10%)
Training courses related to anticholinergic toxicity in the past 12 months		
Yes	0	1 (2.0%)
No	60 (100%)	58 (96.6%)
Maybe	0	1 (1.6%)

**Table 2:** Pre- and post- test scores.

Pre-Test Score Out of 29 (Mean ± Standard Deviation)			*p* Value ^a^
	Intervention group (*n* = 60)	Control group (*n* = 60)	0.987
	19.35 ± 3.56	19.22 ± 3.45	
Post-test score out of 29 (mean ± SD)			
	Intervention group (*n* = 50)	Control group (*n* = 51)	
	22.42 ± 3.812	23.29 ± 3.087	0.208
*p* value ^b^	<0.001	<0.001	

^a^ Mann–Whitney U; ^b^ Wilcoxon signed ranks.

## Appendix A. Content Validity Index of the Knowledge Test Items (33 Items)

**Table:**

	Evaluator								
Question	Exp1	Exp2	Exp3	Exp4	Exp5	Exp6	Exp7	Total # of Experts Voting 3 or 4	I-CVI Score
1	4	2	4	4	1	4	3	5	71.42857143
2	3	4	4	4	2	4	4	6	85.71428571
3	3	4	3	3	1	4	3	6	85.71428571
4	4	4	3	1	4	3	4	6	85.71428571
5	4	4	3	4	4	3	4	7	100
6	3	3	3	3	2	3	4	6	85.71428571
7	4	4	3	3	4	3	4	7	100
8	3	2	2	3	3	3	4	5	71.42857143
9	3	4	3	4	4	4	4	7	100
10	4	3	4	4	1	4	4	6	85.71428571
11	2	3	2	3	4	3	4	5	71.42857143
12a	3	2	3	4	4	3	4	6	85.71428571
12b	3	2	3	4	4	3	4	6	85.71428571
12c	3	3	3	4	2	2	4	5	71.42857143
12d	4	3	4	4	2	4	4	6	85.71428571
13a	3	4	4	4	2	3	4	6	85.71428571
13b	4	4	3	4	4	3	4	7	100
13c	4	4	4	4	3	3	4	7	100
14a	4	4	3	4	2	3	4	6	85.71428571
14b	3	4	4	3	4	3	4	7	100
14c	3	4	4	3	2	3	4	6	85.71428571
14d	4	4	4	4	1	2	4	5	71.42857143
14e	4	4	4	4	1	4	4	6	85.71428571
14f	3	4	4	3	1	3	3	6	85.71428571
15a	4	4	3	4	4	3	4	7	100
15b	3	4	4	4	2	3	4	6	85.71428571
15c	4	4	4	4	4	3	4	7	100
16a	4	4	4	4	4	3	4	7	100
16b	4	4	4	4	1	3	4	6	85.71428571
16c	3	4	3	4	1	3	4	6	85.71428571
16d	4	4	4	4	3	4	4	7	100
17 Rx1	4	4	4	2	3	3	4	6	85.71428571
17 Rx2	4	4	4	2	3	2	4	5	71.42857143

# means number.

## Appendix B. The Final Version of the Knowledge Test (22 Items)

**Table:**

(Q1) Which of the following factors/situations can worsen or intensify anticholinergic side effects? (a) Increasing the daily dose of quetiapine from 100 mg to 200 mg (b) Adding olanzapine to concurrent pseudoephedrine therapy (c) Patient is an older adult (d) All of the above (e) (a) and (b) only
(Q2) Which of the following antidepressant drugs has less anticholinergic activity than paroxetine, which is highly anticholinergic? (a) Citalopram (b) Sertraline (c) Desipramine (d) All of the above (e) (a) and (b) only
(Q3) A patient has been taking baclofen for many years, and is complaining of dry mouth, dry skin and difficulty urinating. Recently tramadol was added to their therapy. Which of the following would you expect to happen? (a) A worsening of dry mouth (b) A worsening of dry skin (c) More difficulty urinating (d) All of the above (e) (a) and (b) only
(Q4) Symptoms of mild anticholinergic toxicity include: (a) Dilated pupils (b) Dry mouth (c) Seizures (d) All of the above (e) (a) and (b) only
(Q5) A patient has taken paroxetine for many years with no reported anticholinergic effects. After 10 years, at the age of 72, the patient starts to notice some dry mouth. Could paroxetine be causing these symptoms now, even though it was not in the past? (a) Yes (b) No
(Q6) Symptoms of anticholinergic effects can occur within: (a) 1–2 h of taking anticholinergic drugs (b) 12–24 h of taking anticholinergic drugs (c) 2–4 days of taking anticholinergic drugs (d) 1–2 weeks of taking anticholinergic drugs
(Q7) A patient taking oxcarbazepine who complains of urinary retention and dry skin is switched to lamotrigine. How would you expect their complaints to be affected by the drug change? (a) Less urinary retention and less dry skin (b) More urinary retention and more dry skin (c) Less urinary retention and more dry skin (d) More urinary retention and less dry skin
(Q8) P.J. is a frail 81-year-old male patient with hypertension and insomnia. His current medication list includes: doxepin 3 mg once daily at bedtime and lisinopril 10 mg once daily. (Q8.a) Which of the following factors put P.J. at higher risk for serious anticholinergic side effects? (a) Age (b) Use of doxepin (c) Hypertension (d) All of the above (e) (a) and (b) only
Today P.J. comes to your pharmacy complaining of dry mouth and urinary retention for the past 3 days. Last week, P.J.’s doctor increased the doxepin dose to 6 mg once daily at bedtime to help address his symptoms of insomnia. P.J. tells you that he has been taking 2 tablets of doxepin 6 mg at bedtime since he could not sleep. (Q8. b) What could you do to help P.J.? (a) Call or fax the prescriber to request a new prescription for a dose reduction of the doxepin dose down to 3 mg once at bedtime. (b) Call or fax the prescriber to recommend stopping the doxepin and switching to trazodone. (c) Refer P.J. to his primary care provider to get a medication to treat urinary retention.
(Q9) M.K., a 52-year-old female patient, comes to the pharmacy to ask for your recommendation for the appropriate dose for loperamide. She has been experiencing mild diarrhea for the past two days. She is not taking any chronic medications or OTC drugs other than acetaminophen for an occasional headache. (Q9.a) Loperamide is classified as a drug with what level of anticholinergic activity? (a) Low anticholinergic activity (b) Moderate/high anticholinergic activity
(Q9.b) M.K. has one or more attributes that put her at risk for serious anticholinergic side effects. (a) True (b) False
(Q9.c) Which measure(s) should you take/recommend to help prevent anticholinergic toxicity with her use of loperamide? (a) Advise M.K. to use the lowest effective dose; not to exceed 16 mg/day (b) Teach M.K. to recognize the signs of anticholinergic side effects and toxicity (c) Follow up with M.K. 1–2 days after starting loperamide to ensure the maximum dose hasn’t been exceeded (d) All of the above (e) (a) and (b) only
(Q10) H.A. a 71-year-old man, comes to your pharmacy complaining of back pain for the past 2 days after lifting a heavy bag. He asks for your advice on taking an OTC muscle relaxant with methocarbamol. After a thorough assessment, you determine that H.A. is not taking any prescribed medications. He only takes diphenhydramine every other night to help him sleep. (Q10.a) Methocarbamol is classified as a drug with what level of anticholinergic activity? (a) Low anticholinergic activity (b) Moderate/high anticholinergic activity
(Q10.b) What would you recommend to help H.A. manage his back pain and minimize the chance of any adverse effects from the medication? (a) Advise H.A. to use a methocarbamol product two tablets every 6 h (b) Advise H.A. to use acetaminophen 500 mg 1 to 2 tablets every 4 to 6 h as needed (c) Advise H.A. to use orphenadrine 100 mg twice daily
(Q11) S.S. a 67-year-old female patient with type 2 diabetes, hypertension and dyslipidemia, comes to the pharmacy for a medication review. Her current medication list includes metformin, lisinopril, atorvastatin, ranitidine, ipratropium and oxybutynin. Current blood pressure 126/73, heart rate 90, HbA1C% 6.7, lipid profile within normal range
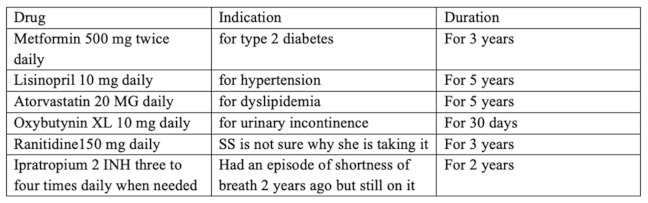
(Q11.a) Which of the following drugs is classified as having moderate/high anticholinergic activity? (a) Oxybutynin (b) Metformin (c) Famotidine (d) Ipratropium
(Q11.b) What other information do you need to collect to better assess for the risk of anticholinergic toxicity? (a) Any over the counter (OTC) drug use (b) Patient’s awareness of the signs of anticholinergic side effects and toxicity (c) How often her conditions are being reassessed by her primary care provider(s) (d) All of the above (e) Only (a) and (b)
(Q11.c) After completing the medication review and assessment of all S.S’s medications, using the resources provided, please write your recommendation/(s) to S.S.’s family doctor, as you would in your practice site. ………………………………………………………………………………………………………………………………………………………………………………………………………………………
(Q12) K.L., a 72-year-old male patient, comes to the pharmacy to pick up his prescription for citalopram. His current medication list includes captopril 12.5 three times daily, risperidone 4 mg daily, ranitidine 150 mg once daily, carbidopa/levodopa 100/25 three times a day, and trazodone 50 mg at bedtime. All his medical conditions are currently stable and he is not complaining of any side effects. He denies taking any over the counter (OTC) medications. (Q12.a) Which of the following factors put K.L. at an increased risk for serious anticholinergic side effects? (a) Age (b) Taking multiple medications (c) History of Parkinson’s disease (d) All of the above (e) (a) and (b) only
Three weeks later, K.L. comes back to the pharmacy with a new prescription, increasing the dose for ranitidine 150 mg to twice daily for his ongoing heartburn. (Q12.b) Could this change in K.L.’s drug regimen increase his risk for anticholinergic side effects? (a) Yes (b) No
(Q12.c) What might you do to help lessen K.L.’s risk for possible anticholinergic side effects? (a) Follow up K.L. 1–2 days after any dose increase in a medication with anticholinergic activity 1 to assess for new side effects (b) Reassess the need for anticholinergic drugs annually (c) Teach K.L. to recognize the signs of anticholinergic side effects and toxicity (d) All of the above (e) (a) and (b) only
13. Based on your clinical judgement, would you fill the following prescriptions? Please select the best answer from the choices provided, then provide a recommendation according to your choice.
Rx 1 a. Fill the prescription as is b. Fill the prescription with patient education if (b) chosen: Please write what would you say to educate the patient c. Contact the prescriber to offer an alternative if (c) chosen: please write your recommendation to the prescriber as you would in your practice site.
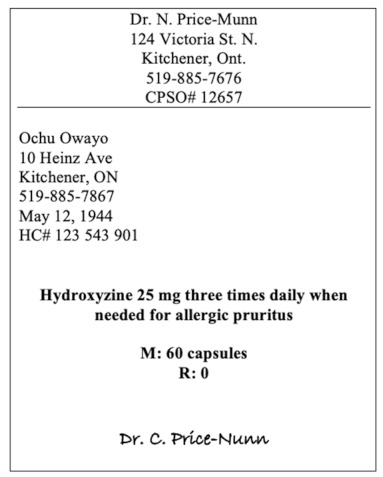
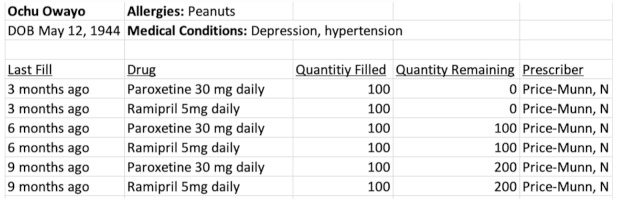
Rx 2 a. Fill the prescription as is b. Fill the prescription with patient education if (b) chosen: Please write what would you say to educate the patient c. Contact the prescriber to offer an alternative if (c) chosen: please write your recommendation to the prescriber as you would in your practice site.
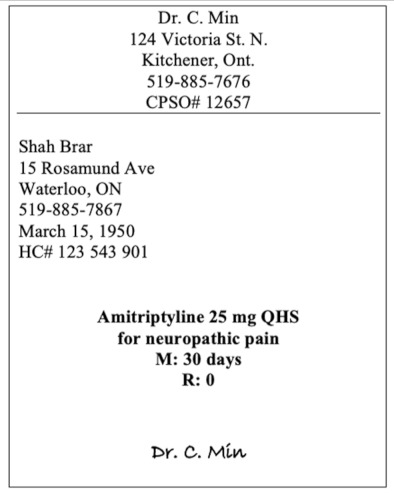
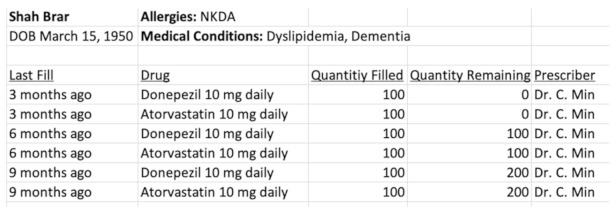

## Appendix C. Subgroup Analysis of Post-Test Knowledge Multiple Choice Questions

**Table:**

Questions	Study Group			
	Intervention Group *n* = 50		Control Group *n* = 51	
	Correct	Incorrect	Correct	Incorrect
Q1	47	3	45	6
Q2	36	14	48	3
Q3	49	1	40	11
Q4	37	13	39	12
Q5	43	7	47	4
Q6	45	5	44	7
Q7	46	4	47	4
Q8.a	5	45	12	39
Q8.b	44	6	45	6
Q9.a	41	9	39	12
Q9.b	39	11	41	10
Q9.c	48	2	45	6
Q10.a	42	8	46	5
Q10.b	48	2	48	3
Q11.a	48	2	49	2
Q11.b	41	9	44	7
Q12.a	4	46	13	38
Q12.b	44	6	49	2
Q12.c	48	2	50	1

## Appendix D. Subgroup Analysis of Post-Test Knowledge Open-Ended Questions

**Table:**

Rx	Study Group					
	Intervention Group *n* = 50			Control Group *n* = 51		
	Fill as is	fill the prescription with education	Contact prescriber to offer an alternative	Fill as is	fill the prescription with education	Contact prescriber to offer an alternative
Rx 1	2	11	37	1	18	32
Rx 2	2	10	38	1	4	46
